# Vitamin D Status in Relation to the Clinical Outcome of Hospitalized COVID-19 Patients

**DOI:** 10.3389/fmed.2022.843737

**Published:** 2022-03-29

**Authors:** Wael Hafez, Husam Saleh, Arun Arya, Mouhamad Alzouhbi, Osman Fdl Alla, Kumar Lal, Samy Kishk, Sara Ali, Srinivasa Raghu, Walaa Elgaili, Wissam Abdul Hadi

**Affiliations:** ^1^NMC Royal Hospital, Abu Dhabi, United Arab Emirates; ^2^The Medical Research Division, Department of Internal Medicine, The National Research Center, Cairo, Egypt

**Keywords:** vitamin D, COVID-19, severity, mortality, United Arab Emirates (UAE), cytokine storm

## Abstract

Coronavirus Disease (COVID-19) is a newly emerged infectious disease that first appeared in China. Vitamin D is a steroid hormone with an anti-inflammatory protective role during viral infections, including SARS-CoV-2 infection, via regulating the innate and adaptive immune responses. The study aimed to investigate the correlation between serum 25-hydroxyvitamin D (25[OH]D) levels and clinical outcomes of COVID-19. This was a retrospective study of 126 COVID-19 patients treated in NMC Royal Hospital, UAE. The mean age of patients was 43 ± 12 years. Eighty three percentage of patients were males, 51% patients were with sufficient (> 20 ng/mL), 41% with insufficient (12–20 ng/mL), and 8% with deficient (<12 ng/mL) serum 25(OH)D levels. There was a statistically significant correlation between vitamin D deficiency and mortality (*p* = 0.04). There was a statistically significant correlation between 25(OH)D levels and ICU admission (*p* = 0.03), but not with the need for mechanical ventilation (*p* = 0.07). The results showed increased severity and mortality by 9 and 13%, respectively, for each one-year increase in age. This effect was maintained after adjustment for age and gender (Model-1) and age, gender, race, and co-morbidities (Models-2,3). 25(OH)D levels (<12 ng/mL) showed a significant increase in mortality by eight folds before adjustments (*p* = 0.01), by 12 folds in Model-1 (*p* = 0.04), and by 62 folds in the Model-2. 25(OH)D levels (< 20 ng/mL) showed no association with mortality before adjustment and in Model-1. However, it showed a significant increase in mortality by 29 folds in Model-3. Neither 25(OH)D levels (<12 ng/mL) nor (< 20 ng/mL) were risk factors for severity. Radiological findings were not significantly different among patients with different 25(OH)D levels. Despite observed shorter time till viral clearance and time from cytokine release storm to recovery among patients with sufficient 25(OH)D levels, the findings were statistically insignificant. In conclusion, we demonstrated a significant correlation between vitamin D deficiency and poor COVID-19 outcomes.

## Introduction

Coronavirus Disease (COVID-19) is a newly emerged infectious disease that first appeared as a series of pneumonia cases in Wuhan, China, by December 2019 (1). The causative organism was identified later and named severe acute respiratory syndrome coronavirus-2 (SARS-CoV-2) (2). On March 11, WHO had announced COVID-19 as a pandemic disease (3). Disease symptoms and manifestation range from influenza-like symptoms, gastrointestinal tract symptoms, acute respiratory distress syndrome (ARDS), multi-organ failure, and death (4, 5).

Vitamin D is a pluripotent steroid hormone with different biological functions involved in bone and calcium metabolism (6). It also regulates innate and adaptive immune responses against respiratory viral infections (7). The role of vitamin D supplementation during viral infections is still controversial.

The protective role of vitamin D during viral infections could be mediated by enhancing the innate immune response by increasing the cellular expression of the antimicrobial peptides and anti-oxidative genes and maintaining the integrity of the respiratory epithelial tight junctions (8). Also, vitamin D modulates the adaptive immune system through the suppression of T helper type-1 (Th1) cells, decreasing the production of pro-inflammatory cytokines and diverting the development of the inflammatory T helper type 17 (Th17) cells to the anti-inflammatory regulatory T cells (T-reg cell). Hence, vitamin D plays an immune-modulatory role during SARS-CoV-2 infection via cytokine modulation, immune cells regulation, and prevention of immune-mediated injury (9).

Vitamin D Deficiency is considered a major health problem worldwide; there are more than one billion individuals worldwide with decreased serum levels of vitamin D (10, 11). A previous randomized clinical trial by Arihiro et al. showed that vitamin D deficiency was associated with increased susceptibility to upper respiratory tract infections (12). While another clinical trial by Aglipay et al. indicated no beneficial effect of vitamin D supplementation to prevent upper respiratory tract infections among children (13).

In the context of COVID-19: several studies reported a correlation between vitamin D deficiency and the risk of infection with SARS-CoV-2 and developing severe COVID-19 outcomes (14, 15). A population-based study conducted in Israel showed an increased prevalence of vitamin deficiency among SAR-CoV-2 positive patients; they also indicated a correlation between plasma 25(OH) D level Deficiency and risk of infection and hospitalization among COVID-19 patients (16). Ilie et al. also reported a significant correlation between serum 25(OH) D levels and SARS-CoV-2 infection and mortality, especially among the elderly in 20 European countries (17). On the other hand, Orchard et al. showed no significant association between vitamin D levels and clinical outcomes of COVID-19 (18).

The current study aimed to investigate the correlation between serum 25(OH)D levels and outcomes of COVID-19.

## Method

### Study Design and Population

The current study was a non-interventional, retrospective study that was carried out on 126 COVID-19 patients treated in NMC Royal Hospital, Khalifa City, Abu Dhabi, UAE, between 8th April 2020 till the end of May 2020. Patients with missing information or chronic liver or kidney disease before contracting COVID-19 were excluded from the study.

A confirmed case of COVID-19 was defined as a positive result real-time reverse transcriptase-polymerase-chain reaction (RT-PCR) assay of nasopharyngeal swab specimens. RT-PCR was performed on The Bio-Rad Cycler PCR, USA, using Solgent's 2019-nCoV Real-Time Reverse Transcription PCR Kit, in line with the manufacturer's instructions. CFX-96 plate reader from Biorad was used for viral detection. A cycle threshold (CT) value above 40 was considered a positive result. This study was conducted according to the Declaration of Helsinki. The study was reviewed and approved by the NMC Central scientific committee reference (NMCHC/CSC/2020/003) and NMC Regional Research Ethics Committee, Abu Dhabi approval reference (NMC/PREC/AUH/2020/ 0016), and Abu Dhabi Health COVID-19 Research Ethics Committee (DOH/CVDC/2020/231).

### Data Collection

The following data were collected from all patients' medical reports: demographic and clinical characteristics, laboratory findings, radiography, treatment, and outcomes were retrieved from the electronic medical records. According to clinical evaluation, X-ray chest and/or chest CT was conducted for all patients at admission and different intervals. The severity of COVID-19 was classified according to WHO COVID-19 Severity guidelines.

### Serum 25(OH)D Analysis

We tested total serum 25(OH)D levels to determine vitamin D status at the time of admission for COVID-19 patients with different disease severity. Serum 25(OH)D levels in the plasma were measured using automated electrochemiluminescence (Atellica IM, Siemens, Germany). Atellica IM VitD assay has a correlation coefficient of 0.95 and a slope of 1.00.1. (Atellica IM VitD, Siemens, Germany). Serum 25(OH)D levels were classified with cut-off value into sufficient (>20 ng/mL), insufficient (12–20 ng/mL), and deficient (<12 ng/mL).

### Data Management and Statistical Analysis

After data collection and verification, all data were entered for statistical analysis using R Software version 3.5.2 (2018-12-20–“Eggshell Igloo,” and the appropriate statistical tests were carried out. Quantitative data with normal distribution were presented as mean ± standard deviation (SD), and range, when normal distribution was violated, data were presented as median and interquartile range. Qualitative data were presented as frequency (n) and percentage (%). Shapiro test was used to verify the normality of distribution, and logarithmic transformation had been applied if appropriate. Chi-square test was used to determine the association between serum 25(OH)D levels and severity, mortality, intensive care unit (ICU) admission, and need for mechanical ventilation among COVID-19 patients.

The logistic regression model was performed to determine the independent association of vitamin D sufficiency with the severity of COVID-19, mortality, and factors that were significant on the univariate analysis. The linear regression model was conducted to estimate the correlation between serum 25(OH)D levels and immune-inflammatory response biochemical markers using the Spearman correlation coefficient (r). The Kruskal Wallis test was used to analyze the association between serum 25(OH)D levels and immune-inflammatory response markers. The confidence interval was set to 95%, and the margin of error accepted was set to 5%. So, the *p*-value was considered significant as the following: *P* > 0.05: non-significant (NS), *P* < 0.05: significant (S), and *P* < 0.01: highly significant (HS).

## Results

### Characteristics of COVID-19 Patients According to Disease Severity and Mortality

The study included 126 COVID-19 patients with a mean age of 43 ± 12 years. There was a strong correlation between disease severity and mortality with increased age, the mean age of non-severe patients was 39 ± 10 years old, while the mean age of severe patients was 49 ± 12 years old. Also, the mean age was 42 ± 11 and 59 ± 12 years old among discharged and dead COVID-19 patients, respectively (*p* < 0.001). 83% of patients were males, while 75% of patients were Asian and showed significantly decreased mortality prevalence compared to other races.

Regarding co-morbidities, 18% of hypertensive patients showed a significantly increased mortality compared to normotensive ones (*p* = 0.001). Seventeen (14%) patients were admitted to the ICU, all of them had severe symptoms, and nine (53%) of them had died; there was a statistically significant correlation between ICU admission and mortality and severity among COVID-19 patients (*p* < 0.001).

In our study, 51% of patients were with sufficient serum 25(OH)D levels (> 20 ng/mL), 41% were with insufficient levels (12–20 ng/mL), and 8% were with deficient serum 25(OH)D levels (<12 ng/mL). There was a statistically significant correlation between vitamin D deficiency and mortality due to COVID-19 (*p* = 0.04).

Regarding immune-inflammatory response biomarkers, there were significantly increased CRP, Ferritin, LDH, Fibrinogen, and D-dimer. At the same time, there was a significant decrease in the lymphocytic count among severe and dead patients (Table 1).

**Table 1:** Characteristics of COVID-19 patients according to disease severity and mortality.

			**Severity**			**Mortality**		
		***N***	**Non-severe**	**Severe**	***P***	**Alive**	**Died**	***P***
Total		126	81	45		117	9	
Age (years)	Mean ± SD	43 ± 12	39 ± 10	49 ± 12	<0.001	42 ± 11	59 ± 12	<0.001
Sex	Female	21 (17%)	16 (76.2%)	5 (23.8%)	0.32	19 (90.5%)	2 (9.5%)	0.64
	Male	105 (83%)	65 (62%)	40 (38%)		98 (93%)	7 (7%)	
Race	Asian	95 (75%)	62 (65%)	33 (35%)	0.63	92 (97%)	3 (3%)	0.009
	Black	5 (4%)	4 (80%)	1 (20%)		4 (80%)	1 (20%)	
	White	26 (21%)	15 (58%)	11 (42%)		21 (81%)	5 (19%)	
BMI (kg/m^2^)	Mean ± SD	28 ± 6	27 ± 5	30 ± 6	0.006	28 ± 5	31 ± 9	0.27
Obesity (BMI>30 kg/m^2^)	Not-obese	88 (74%)	61 (69%)	27 (31%)	0.03	82 (93%)	6 (7%)	0.7
	Obese	31 (26%)	14 (45%)	17 (55%)		28 (90%)	3 (10%)	
Vitamin D level (ng/mL)
<12	Deficient	10 (8%)	5 (50%)	5 (50%)	0.32	7 (70%)	3 (30%)	0.04
12–20	Insufficient	52 (41%)	37 (71%)	15 (29%)		50 (96%)	2 (4%)	
> 20	Sufficient	64 (51%)	39 (61%)	25 (39%)		60 (94%)	4 (6%)	
HTN	No	103 (82%)	70 (68%)	33 (32%)	0.11	100 (97%)	3 (3%)	0.001
	Yes	23 (18%)	11 (48%)	12 (52%)		17 (74%)	6 (26%)	
DM	No	100 (79%)	69 (69%)	31 (31%)	0.05	95 (95%)	5 (5%)	0.09
	Yes	26 (21%)	12 (46%)	14 (54%)		22 (85%)	4 (15%)	
CVS	No	122 (97%)	79 (65%)	43 (35%)	0.62	114 (93%)	8 (7%)	0.26
	Yes	4 (3%)	2 (50%)	2 (50%)		3 (75%)	1 (25%)	
ICU admission	No	109 (87%)	81 (74%)	28 (26%)	<0.001	109 (100%)	0 (0%)	<0.001
	Yes	17 (14%)	0 (0%)	17 (100%)		8 (47%)	9 (53%)	
Ventilation	No	82 (65%)	80 (98%)	2 (2%)	<0.001	82 (100%)	0 (0%)	<0.001
	Invasive	9 (7%)	0 (0%)	9 (100%)		0 (0%)	9 (100%)	
	Low flow O2	18 (14%)	1 (6%)	17 (94%)		18 (100%)	0 (0%)	
	Non-invasive	17 (14%)	0 (0%)	17 (100%)		17 (100%)	0 (0%)	
Platelets (× 10^9^/L)	Mean ± SD	324 ± 140	304 ± 112	359 ± 176	0.22	329 ± 143	247 ± 47	0.13
Lymphocyte (× 10^9^/L)	Mean ± SD	26 ± 13	31 ± 11	18 ± 11	<0.001	28 ± 12	9 ± 5	<0.001
CRP (mg/L)	Mean ± SD	69 ± 82	34 ± 51	132 ± 91	<0.001	59 ± 70	198 ± 116	<0.001
Ferritin (ng/mL)	Mean ± SD	804 ± 1,196	328 ± 312	1,662 ± 1,648	<0.001	666 ± 992	2,603 ± 2,038	<0.001
LDH (U/L)	Mean ± SD	381 ± 369	257 ± 101	604 ± 538	<0.001	321 ± 155	1,162 ± 1,018	<0.001
Fibrinogen (mg/dL)	Mean ± SD	554 ± 203	460 ± 163	724 ± 152	<0.001	535 ± 190	811 ± 205	0.0009
D-Dimer (μg/mL)	Mean ± SD	3 ± 7	1 ± 3	6 ± 10	<0.001	2 ± 5	17 ± 11	<0.001

*Data are presented as range, and mean ± SD for continuous variables, and n and % for categorical variables*.

### Association Between Serum 25(OH)D Levels and Need of Mechanical Ventilation, and ICU Admission

There were three (30%) patients of deficient vitamin D group on invasive ventilation and two (20%) on non-invasive ventilation. Whereas 39 (61%) patients of the sufficient vitamin D group and 38 (73%) patients of the insufficient vitamin D group did not need exogenous ventilation (*p* = 0.07). Regarding ICU admission, four (40%) patients with deficient vitamin D group were admitted to the ICU compared to nine (14%) patients in the sufficient vitamin D group (*p* = 0.03) (Table 2).

**Table 2:** Association between serum vitamin D levels to ventilation, ICU admission.

		**Ventilation**					**ICU admission**		
**Vitamin D level (ng/mL)**		**No**	**Invasive**	**Low flow O_**2**_**	**Non-invasive**	***P* value**	**No**	**Yes**	***P* value**
	Count (N)	82	9	18	17		109	17	
< 12	Deficient	5 (50%)	3 (30%)	0 (0%)	2 (20%)	0.07	6 (60%)	4 (40%)	0.03
12–20	Insufficient	38 (73%)	2 (4%)	5 (10%)	7 (14%)		48 (92%)	4 (8%)	
>20	Sufficient	39 (61%)	4 (6%)	13 (20%)	8 (13%)		55 (86%)	9 (14%)	

*Data presented as n and %*.

### Predictors for COVID-19 Severity and Mortality Using Logistic Regression Analysis

The un-adjusted logistic regression model showed increased severity and mortality due to COVID-19 by about 9%, and 13%, respectively for each one-year increase in age (OR = 1.09, 95%CI: (1.05–1.14) *p* < 0.001), (OR = 1.13, 95%CI: (1.06–1.22), *p* < 0.001), for severity and mortality, respectively (Tables 3, 4).

**Table 3:** Predictors for COVID-19 severity using univariate and multivariate logistic regression models.

			Model 1^*^		Model 2^**^	
	**Unadjusted model**		**Adjusted**		**Adjusted**	
	**OR (95%CI)**	***P* value**	**OR (95%CI)**	***P* value**	**OR (95%CI)**	***P* value**
Age	1.1 (1.1–1.14)	<0.001	1.09 (1.05–1.14)	<0.001	1.09 (1.04-1.14)	0.001
Male	2 (0.71–6.4)	0.22	2 (0.6–7.02)	0.32	2 (0.58-7.5)	0.30
Obese(BMI > 30 kg/m^2^)	3 (1.2–6.5)	0.02				
25(OH)D < 12 ng/mL	2 (0.5–7.2)	0.33	2 (0.4–7.5)	0.46	2 (0.4-7.7)	0.46
25(OH)D < 20 ng/mL	0.7 (0.4–1.5)	0.43	0.96 (0.4–2.3)	0.92	0.98 (0.4-2.4)	0.96
HTN	2 (0.9–5.9)	0.07			0.91 (0.3-2.95)	0.87
DM	3 (1.1–6.4)	0.03			1.4 (0.5-4)	0.51
CVS	2 (0.2–15.8)	0.55			0.96 (0.08-11.2)	0.98
Platelets (× 10^9^/L)	1 (1–1.01)	0.04				
Lymphocyte (× 10^9^/L)	0.9 (0.86–0.94)	<0.001				
CRP(mg/L)	1.02 (1.01–1.03)	<0.001				
Ferritin (ng/mL)	1 (1.00–1.01)	<0.001				
LDH(U/L)	1.01 (1.01–1.02)	<0.001				
Fibrinogen (mg/dL)	1.01 (1.01–1.01)	<0.001				
D–Dimer (μg/mL)	1.2 (1.06–1.37)	0.01				

* *In model 1: the two cut off levels of 25(OH)D had been adjusted to each other and adjusted for age and gender also, while*

** *in model 2: the two cut off levels of 25(OH)D had been adjusted to each other and also adjusted for age, gender, and comorbidities*.

**Table 4:** Significant predictors of mortality univariate and multivariate logistic regression models.

			Model 1^*^		Model ^**^		Model 3^***^	
	**Unadjusted model**		**Adjusted**		**Adjusted**		**Adjusted**	
	**OR (95%CI)**	***P* value**	**OR (95%CI)**	***P* value**	**OR (95%CI)**	***P* value**	**OR (95%CI)**	***P* value**
Age	1.13 (1.06–1.2)	<0.001	1.17 (1.1–1.3)	0.001	1.15 (1.04–1.3)	0.02	1.18 (1.06–1.4)	0.01
Male	0.68 (0.15–4.8)	0.64	0.47 (0.06–4.7)	0.48	0.2 (0.01–3)	0.22	0.08 (0.00–1.3)	0.09
Race (Asian)	0.14 (0.03–0.6)	0.01			0.37 (0.02–6.3)	0.46	0.30 (0.02–4.5)	0.37
Race (Black)	1.05 (0.05–9.4)	0.97			1.36 (0.01–106)	0.89	0.57 (0.01–36.2)	0.79
Obese(BMI > 30 kg/m^2^)	1.5 (0.3–6)	0.61						
25(OH)D < 12 ng/mL	8 (1.4–37.5)	0.01	12 (1.3–171.6)	0.04	62(3.9–5098)	0.02		
25(OH)D < 20 ng/mL	1.32 (0.33–5.55)	0.69	2 (0.2–28)	0.47			29 (1.9–1507)	0.04
HTN	12 (2.8–60)	0.001			31(2.7–1027)	0.02	29 (2.7–811)	0.02
DM	3.5 (0.8–14)	0.08			0.85 (0.09–8.3)	0.88	0.95 (0.11–7.3)	0.96
CVS	5 (0.22–42)	0.2			0.36 (0.00–12)	0.60	1.32 (0.03–42.1)	0.88
Platelets (× 10^9^/L)	0.99 (0.99–1)	0.10						
Lymphocyte (× 10^9^/L)	0.76 (0.63–0.87)	0.001						
CRP (mg/L)	1.01 (1.01–1.02)	<0.001						
Ferritin (ng/mL)	1.00 (1.00–1.00)	0.004						
LDH(U/L)	1.01 (1.00–1.01)	0.001						
Fibrinogen (mg/dL)	1.01 (1.00–1.01)	0.001						
D-Dimer (μg/mL)	1.16 (1.09–1.27)	<0.001						

* *In model 1: the two cut off levels of 25(OH)D had been adjusted to each other and adjusted for age and gender also*,

** *in model 2: the cut off level 25(OH)D < 12 ng/mL had been adjusted for age, gender, race, and comorbidities while*

*** *in model 3: the cut off level 25(OH)D < 20 ng/mL had been adjusted for age, gender, race, and comorbidities*.

Gender was not a significant risk factor for disease mortality. However, Diabetes also increased disease severity by about three times (OR = 3, 95%CI: (1.1–6.4), *p* = 0.03), while hypertension increased odds of mortality by about 12 times (OR = 12, 95%CI: (2.8–60), *p* = 0.001) (Tables 3, 4).

Regarding immune-inflammatory response biomarkers, increasing Platelets Count, CRP, Ferritin, LDH, Fibrinogen, and D-Dimer levels showed a significant increase in disease severity. While only CRP elevation, Ferritin, LDH, Fibrinogen, and D-Dimer increased mortality risk significantly (Tables 3, 4).

However, increasing Lymphocyte count showed 10% significant decrease in COVID-19 severity, and 24% decrease in mortality [OR = 0.90, 95%CI: (0.86–0.94), *p* < 0.001], [OR = 0.76, 95%CI: (0.63–0.87), *p* = 0.001], respectively (Tables 3, 4).

Neither serum 25(OH)D levels (<12 ng/mL) nor (< 20 ng/mL) were significant as risk factors for severity. On the other hand, the un-adjusted odds of mortality increased significantly among patients with serum 25(OH)D levels (<12 ng/mL) compared with patients with higher serum 25(OH)D levels [OR = 7.86, 95%CI: (1.43–37.49), *p* = 0.01] (Tables 3, 4).

Then we adjusted the logistic logistics model for age and gender only (Model-1), and in (Model-2), only the 25(OH)D < 12 ng/mL cutoff point had been adjusted for age, gender, race, and comorbidities while in (Model-3), only the cutoff point 25(OH)D < 20 ng/mL had been adjusted for age, gender, race, and comorbidities. Based on both models, the results showed that age was still a significant risk factor for disease severity [Model-1: [OR = 1.09, 95%CI: (1.05–1.14), *p* < 0.001], Model-2: (OR = 1.09, 95%CI: (1.04–1.14), *p* = 0.001)]. While diabetes lost its significance after adjustment (Table 3).

Age also remained a significant risk factor for mortality in the three models [Model-1: (OR = 1.17, 95%CI:(1.1–1.3), *p* = 0.001), Model-2: (OR = 1.15, 95%CI: (1.04–1.3), *p* = 0.02), Model-3: (OR=1.18, 95%CI: (1.06-1.4), *p* = 0.01)]. Also, serum 25(OH)D levels (<12 ng/mL) showed significant increase in mortality by 12 folds in Model-1: [OR = 12, 95%CI: (1.3–171.6), *p* = 0.04]. Also showed a significant increase in mortality by about 62 folds in Model-2: (OR = 62, 95%CI: (3.9–5098), *p* = 0.02]. While serum 25(OH)D levels (< 20 ng/mL) did not show any significant association with the disease mortality before adjustment and in Model-1 however, it showed a significant increase in mortality by about 29 folds in Model-3: [OR= 29, 95%CI: (1.9-1507), *p* = 0.04] (Table 4). Regarding co-morbidities, Hypertension significantly increased mortality by 31 folds and 29 folds in Model-2 and Model-3, respectively compared to normotensive patients [OR=31, 95%CI: (2.7–1027), *p* = 0.02], [OR = 29, 95%CI: (2.7–811), *p* = 0.02) respectively] (Table 4).

### The Association Between Serum 25(OH)D Levels and Immune-Inflammatory Response Markers

**Figure 1:**
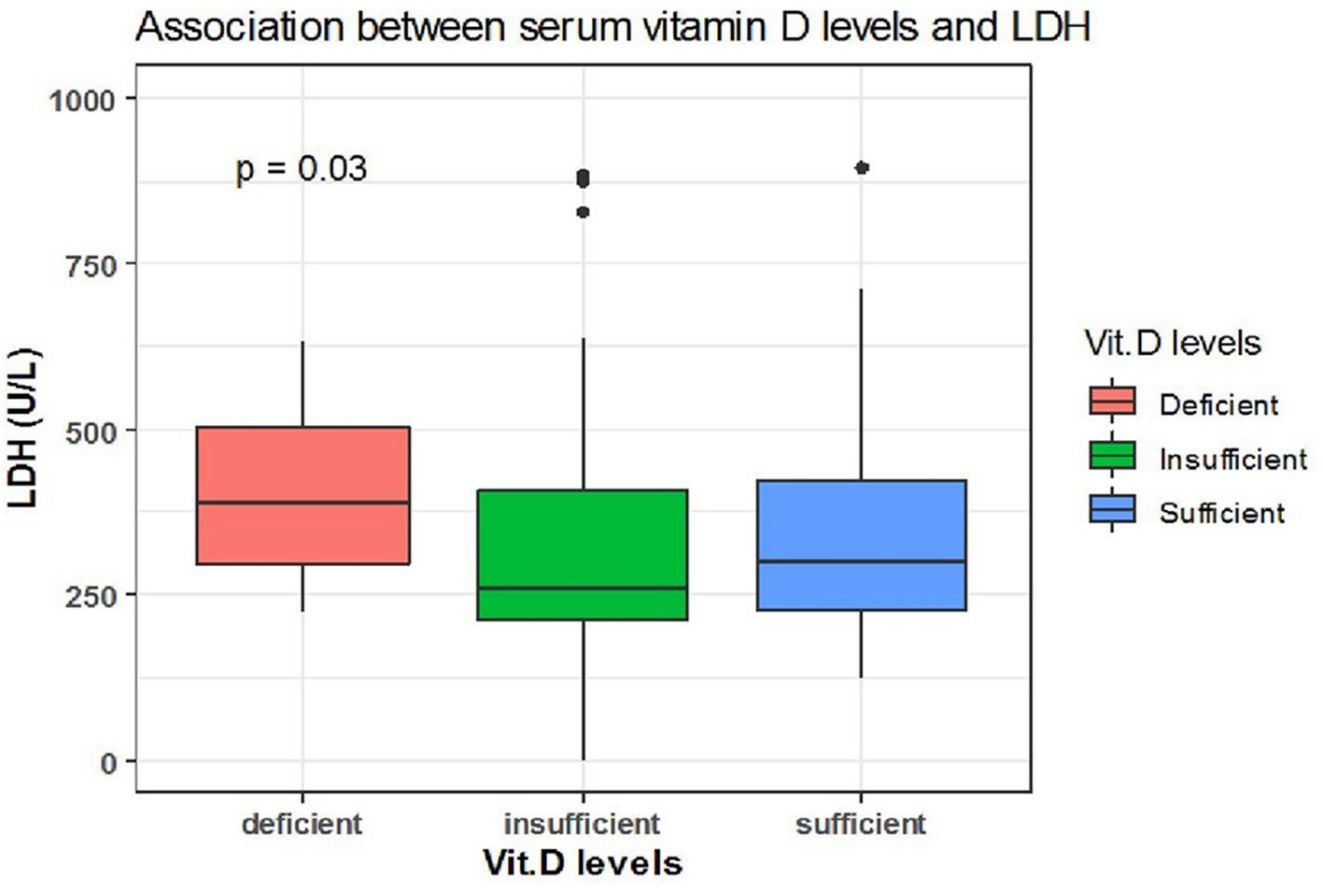
The association between serum 25(OH)D levels (sufficient, insufficient, deficient) and LDH (U/L).

**Figure 2:**
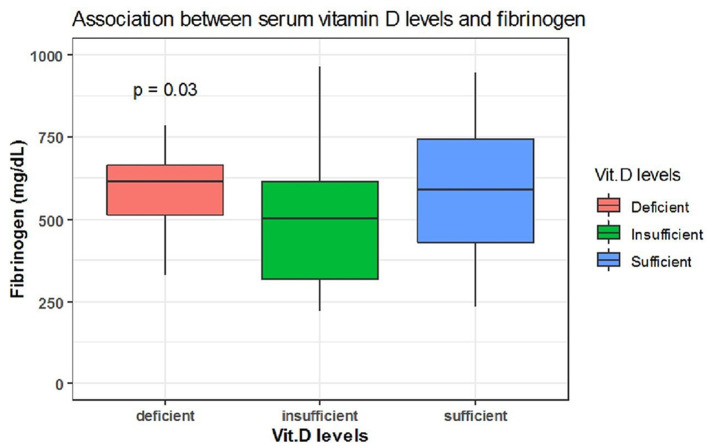
The association between serum 25(OH)D levels (sufficient, insufficient, deficient) and fibrinogen (mg/dL).

**Figure 3:**
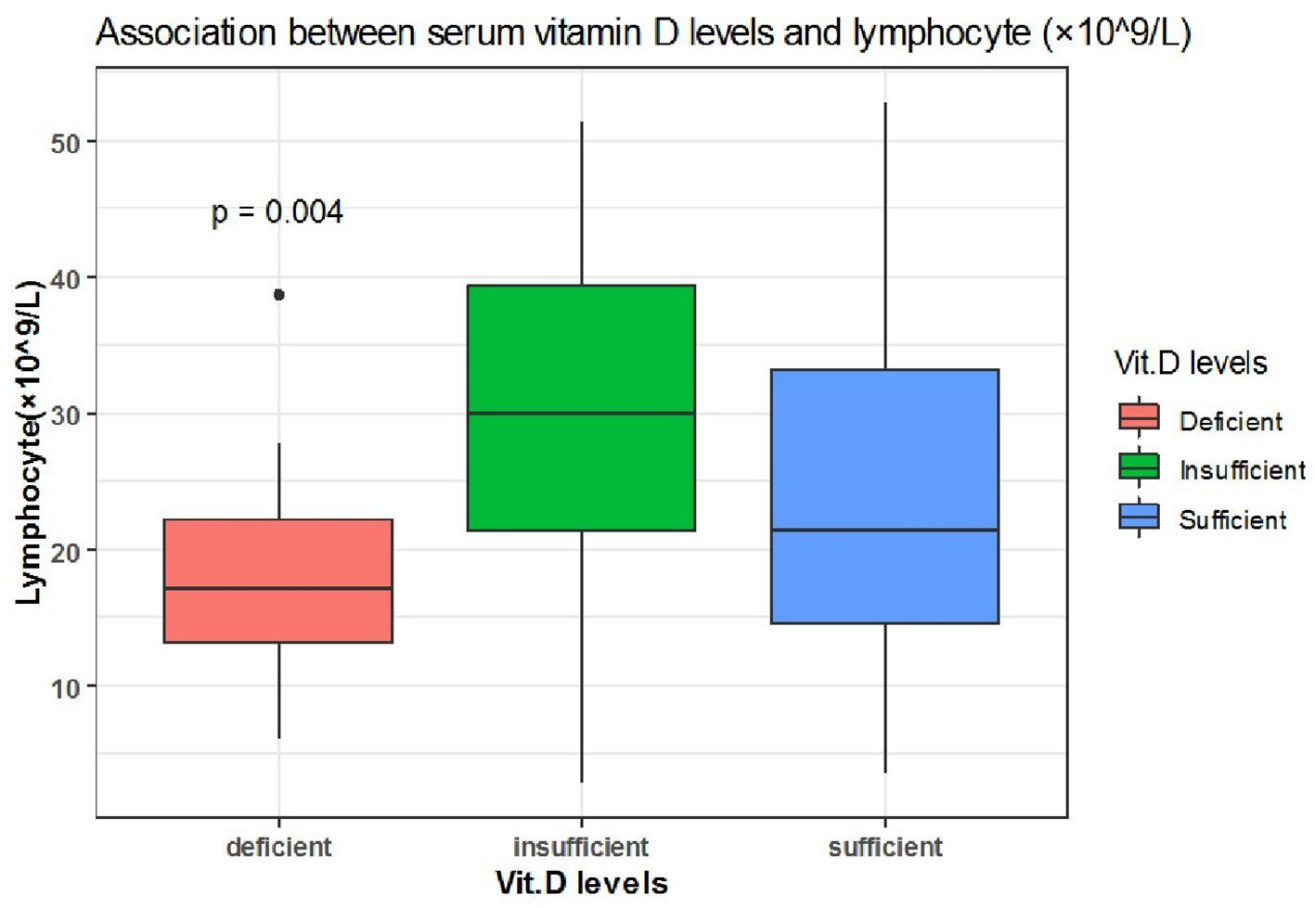
The association between serum 25(OH)D levels (sufficient, insufficient, deficient) and lymphocyte (× 10^9^/L).

Spearman correlation coefficient analysis showed that each one-unit increase in serum 25(OH)D levels were significantly associated with a 1.5 increase in platelets count (× 10^9^/L) (*p* = 0.03) (Table 5). While, the Kruskal Wallis test showed significantly increased LDH (U/L) and Fibrinogen (mg/dL) levels (*p* = 0.03, *p* = 0.03, respectively) among patients with vitamin D deficiency compared to patients with sufficient and insufficient serum 25(OH)D levels (Table 6, Figures 1, 2). There was also a significant association between decreased lymphocytic count(× 10^9^/L) in patients with deficient serum 25(OH)D levels (<12 ng/mL) compared to patients with higher serum 25(OH)D levels (*p* = 0.004) (Table 6, Figure 3).

**Table 5:** Linear regression model investigating the correlation between serum 25(OH)D levels and immune -inflammatory response biochemical markers.

**Immune-inflammatory response markers**	**Regression coefficient (ß) (95%CI)**	**Spearman coefficient (r)**	***P* value**
Platelets (× 10^9^/L)	1.5 (0.2 to 2.8)	0.17	0.03
Lymphocyte (× 10^9^/L)	−0.01 (−0.14 to 0.1)	−0.04	0.82
CRP (mg/L)	−0.3 (−1.1 to 0.5)	−0.02	0.46
Ferritin (ng/mL)	0.6 (−11 to 12)	−0.03	0.91
LDH (U/L)	−1.8 (−5.4 to 1.7)	−0.04	0.31
Fibrinogen (mg/dL)	0.95 (−1 to 2.9)	0.12	0.34
D-Dimer (μg/mL)	−0.02 (−0.1 to 0.05)	0.04	0.6

**Table 6:** Comparative analysis of the association between serum 25(OH)D levels and immune -inflammatory response markers.

**Immune-inflammatory response markers**		**Serum vitamin D levels (ng/mL)**			***P* value**
		**Deficient < 12**	**Insufficient 12–20**	**Sufficient > 20**	
Platelets (× 10^9^/L)	Median (IQR)	248 (124)	274 (185)	298 (176)	0.14
Lymphocyte (× 10^9^/L)	Median (IQR)	17 (9)	30 (18)	21 (19)	0.004
CRP (mg/L)	Median (IQR)	92 (90)	22 (84)	39 (102)	0.35
Ferritin (ng/mL)	Median (IQR)	1,015 (890)	398 (777)	451 (830)	0.25
LDH (U/L)	Median (IQR)	460 (307)	258 (197)	307 (205)	0.03
Fibrinogen (mg/dL)	Median (IQR)	621 (173)	506(306)	588 (317)	0.03
D-Dimer (μg/mL)	Median (IQR)	0.7 (16)	0.4 (0.6)	0.4 (0.8)	0.12

### Time Till Viral Clearance Among Study Population

**Figure 4:**
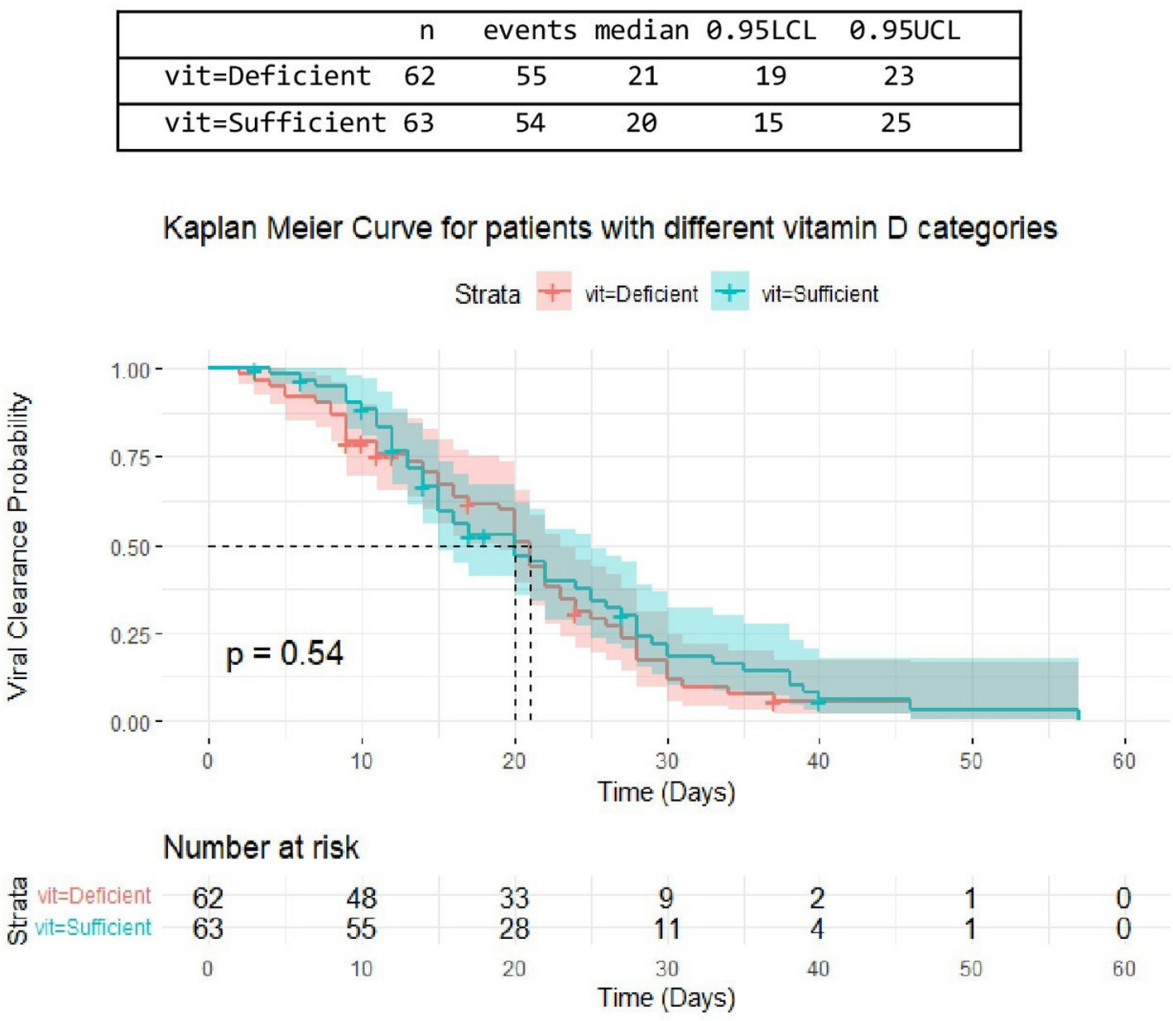
The association between serum 25(OH)D levels (sufficient, deficient) and time untill viral clearance.

There was no difference in time till viral clearance among patients with different serum 25(OH)D levels, despite being a little shorter in patients with sufficient serum 25(OH)D levels. From 62 patients with deficient serum 25(OH)D levels, the median time till viral clearance among 33 patients was [median= 21 days, 95% CI: (19-23)]. From a total of 63 patients with sufficient serum 25(OH)D levels, the median time till viral clearance among 28 patients was [median = 20 days, 95% CI: (15-25)] (Figure 4).

### Association Between Time From Cytokine Release Storm (CRS) Till Discharge and Serum 25(OH)D Levels

**Figure 5:**
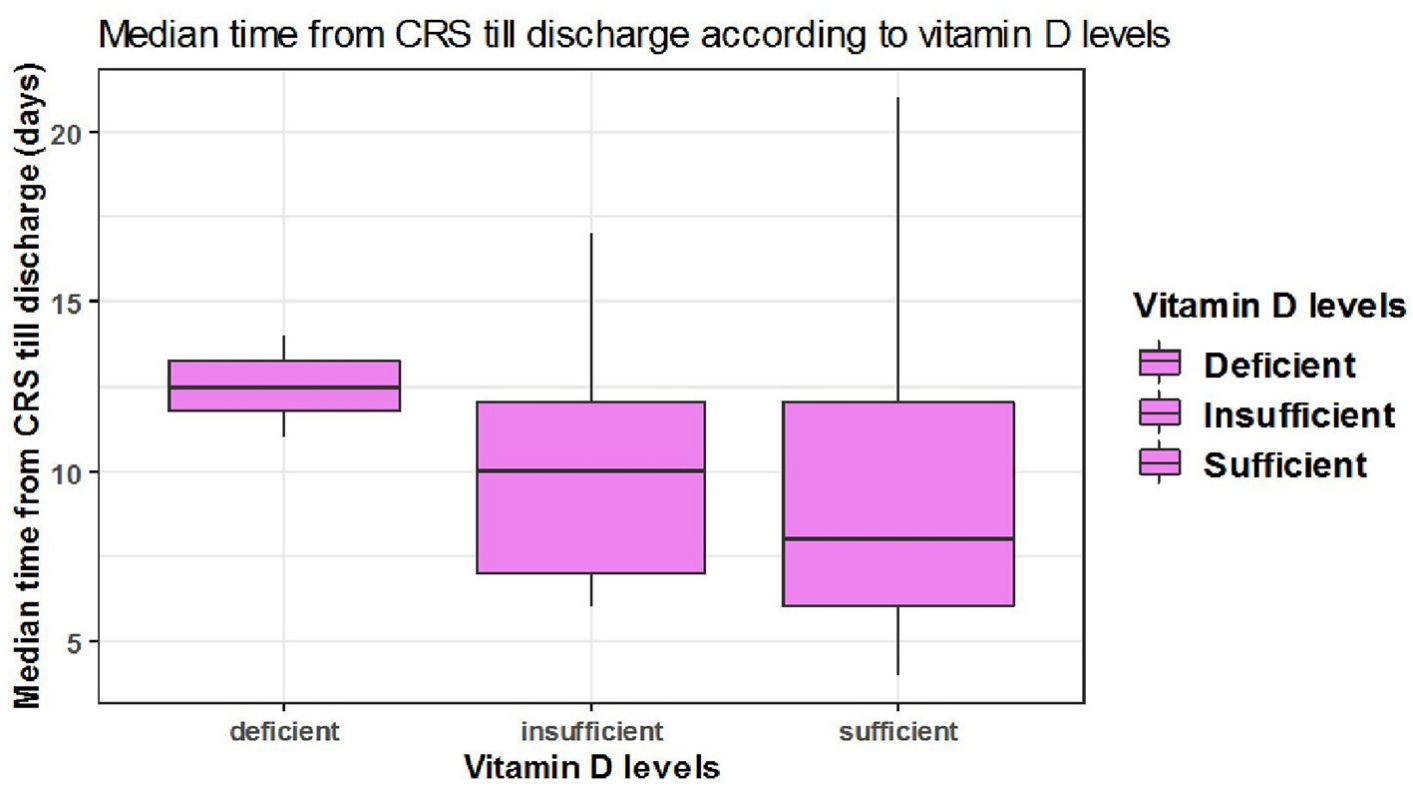
The association between serum 25(OH)D levels (sufficient, insufficient, deficient) and median time CRS till discharge in days.

The median time from CRS to discharge was shorter among patients with sufficient serum 25(OH)D levels however, this difference was not statistically significant. Out of 36 discharged patients who had developed cytokine storm, the median time from CRS till discharge among two patients with deficient, 13 patients with insufficient, and 21 patients with sufficient serum 25(OH)D levels were {[median = 12 days, 95% CI: (12,13)], [median = 10 days, 95% CI: (7-12)], [median = 8 days 95% CI: (6-12)], respectively} with no significant difference between the three categories (*P* = 0.3) (Table 7, Figure 5).

**Table 7:** The association between serum 25(OH)D levels and median time from CRS till discharge.

**Characteristic**	**Deficient**	**Insufficient**	**Sufficient**	***p*-value^b^**
	***N* = 2^a^**	***N* = 13^a^**	***N* = 21^a^**	
Time from CRS onset till fitness for discharge	12 (12,13)	10 (7,12)	8 (6,12)	0.3

a *Median (IQR)*.

b *Wilcoxon rank-sum test. Only severe COVID patients are included*.

### Association Between the Radiological Findings and Serum 25(OH)D Levels

**Figure 6:**
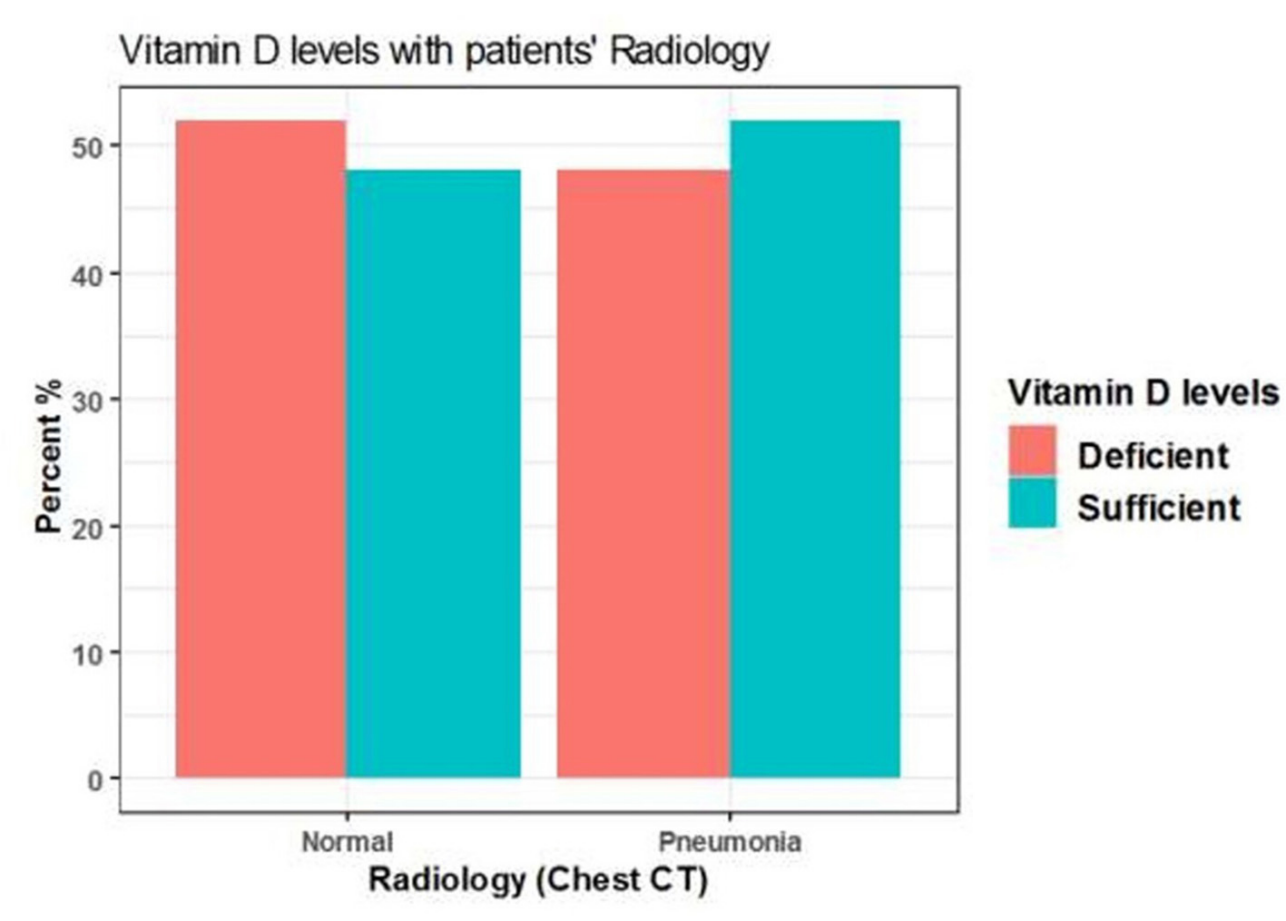
The association between serum 25(OH)D levels (sufficient, deficient) and radiological findings of the patients.

Among 125 patients, 98 included COVID-19 patients were suffering from pneumonia and 27 patients showed no radiological evidence of pneumonia. Pneumonia was presented in 48 (48 %) patients with insufficient serum 25(OH)D levels (<20 ng/mL) and also presented in 50 (51%) patients with sufficient serum 25(OH)D levels (≥20 ng/mL). However, there was no significant difference between serum 25(OH)D levels in patients with or without pneumonia (*P* = 0.8) (Table 8, Figure 6).

**Table 8:** The association between serum 25(OH)D levels (sufficient, deficient) and radiological findings of the patients.

**Characteristic**	**Normal**	**Pneumonia**	***p*-value^b^**
	***N* = 27^a^**	***N* = 98^a^**	
Vitamin D categories			0.8
Insufficient (< 20 ng/mL)	14 (52%)	48 (49%)	
Sufficient (≥ 20 ng/mL)	13 (48%)	50 (51%)	

a *n (%)*.

b *Pearson's Chi-squared test*.

**Figure 7:**
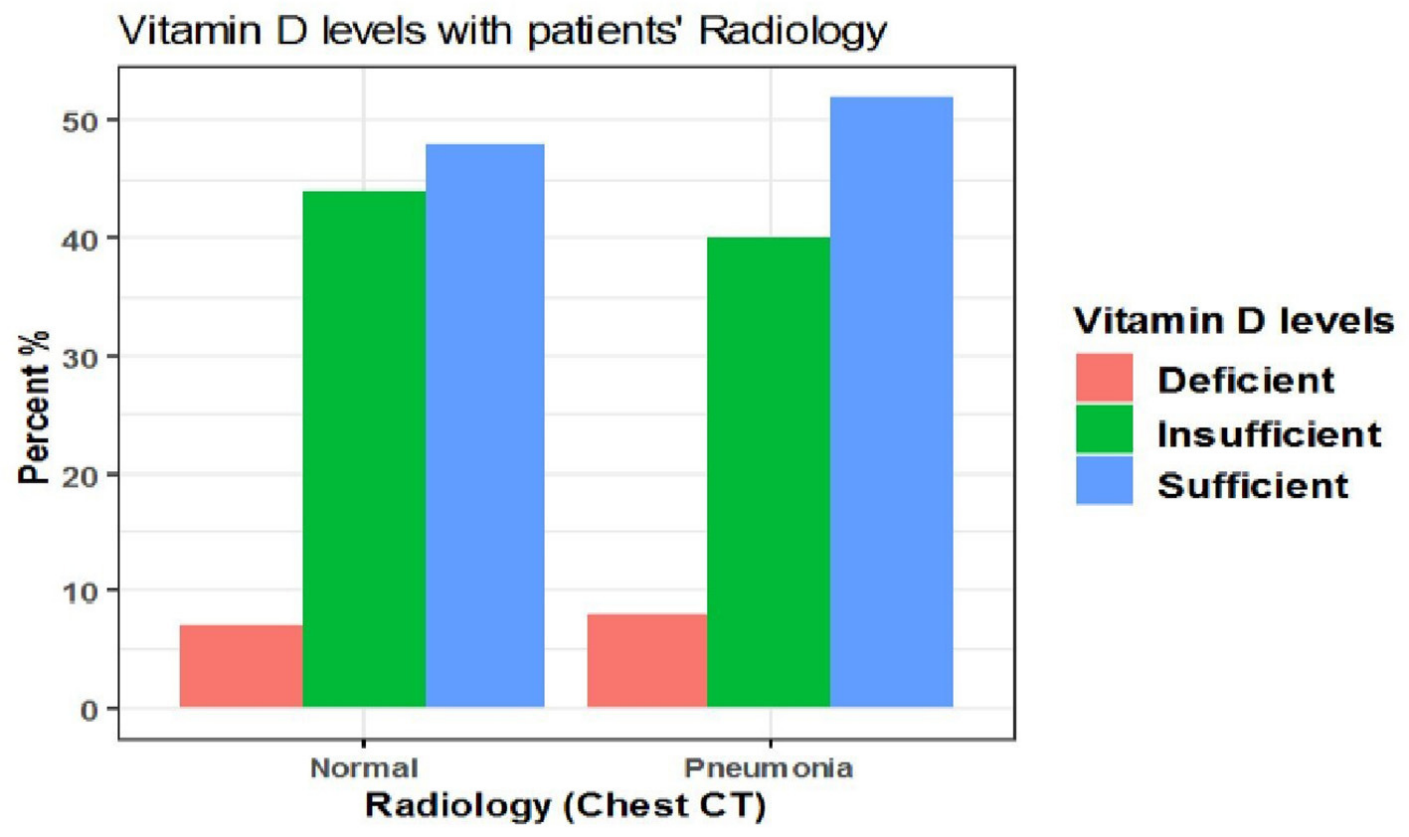
The association between serum 25(OH)D levels (deficient, insufficient, sufficient) and radiological findings of the patients.

The difference also was not significant when vitamin D cut-off values were categorized into deficient (<12 ng/mL), insufficient (12–20 ng/mL), and sufficient (>20 ng/mL) (*p* = 0.9) (Table 9, Figure 7).

**Table 9:** The association between serum 25(OH)D levels (deficient, insufficient, sufficient) and radiological findings of the patients.

**Characteristic**	**Normal**	**Pneumonia**	***p*-value^b^**
	***N* = 27^b^**	***N* = 98^b^**	
Vitamin D categories			>0.9
Deficient (< 12 ng/mL)	2 (7.4%)	8 (8.2%)	
Insufficient (12–20 ng/mL)	12 (44%)	40 (41%)	
Sufficient (> 20 ng/mL)	13 (48%)	50 (51%)	

a *n (%)*.

b *Fisher's exact test*.

## Discussion

The current study investigated the correlation between serum 25(OH)D levels and COVID-19 prognosis among patients treated at NMC Royal Hospital in UAE. We found a statistically significant correlation between vitamin D deficiency, ICU admission, and in-hospital mortality due to COVID-19. However, the need for mechanical ventilation was not correlated. Several studies had also reported a correlation between low serum 25(OH)D levels and poor COVID-19 outcomes.

A study by De Smet et al. showed a high prevalence (59%) of hospitalization among COVID-19 with serum 25(OH)D levels <20 ng/mL at the time of admission, especially among male patients and with increased age. They also reported an independent correlation between low 25(OH)D levels and mortality due to COVID-19 (OR = 3.87) (19). Radujkovic et al. also showed a strong correlation between vitamin D deficiency and the need for mechanical ventilation and/or mortality (HR 6.12 and 14.73, respectively) (20). These observations are consistent with our findings as we found a significant association between vitamin D deficiency and mortality (*p* = 0.036). Another meta-analysis of 1,368 COVID-19 positive patients by Munshi et al. also reported that patients with poor prognosis had significantly decreased mean serum levels of vitamin D (21).

However, the need for mechanical ventilation was not significantly associated with vitamin D deficiency (*p* = 0.073), which is in contrast to the findings of several reports (20, 22, 23). This inconsistency suggests several confounding factors or deficiency of several nutrients affects this correlation.

Maghbooli et al. showed an independent association between vitamin D sufficiency and decreased severity due to COVID-19; the authors showed no association between vitamin D deficiency and ICU admission. In contrast to our findings, the logistic regression model indicated no association between circulating 25(OH)D levels and the severity of COVID-19 (24). But, the authors considered a cut-off point of 30 ng/mL for vitamin D sufficiency, while in our study, vitamin D sufficiency was defined using a cut-off value of >20 ng/mL. While Rhodes et al. showed lower mortality rates in all countries below 35 degrees due to sufficient exposure to sunlight appropriate for vitamin D synthesis (14).

In UAE, despite that, there is abundant sunlight, but most of the population suffers from hypovitaminosis or vitamin D deficiency. A retrospective study by Sridhar et al. reported that among 425 subjects, there were 48.9, 33.2, and 14.8% subjects with vitamin D deficiency, severe deficiency, and insufficiency, respectively (25).

Alsafar et al. studied the correlation between serum 25(OH)D levels and COVID-19 outcomes, and the findings of the study were similar to ours in different aspects. The authors showed a statistically significant correlation between the severity of COVID-19 and age, obesity, and the presence of diabetes mellitus. They also reported that serum 25(OH) D levels <12 ng/mL had a significant association with an elevated risk of severe COVID-19 infection and death (26).

Karahan et al. had indicated a statistically significant correlation between the severity of COVID-19 and diabetes mellitus, which is similar to our observation. Still, they also showed a strong correlation between hypertension and disease severity, which was not observed among our cohort. The authors also reported a significant correlation between COVID-19 severity and several biochemical parameters, including CRP, lymphocyte count, and non-significant association with platelet count; they also indicated a strong correlation between mortality and vitamin D deficiency in line with our observations. On the other hand, Karahan and Katkat showed a significant correlation between serum 25(OH) D levels and disease severity, which opposed our findings (27). In our study, age was strongly correlated with severity and mortality due to COVID-19; logistic regression analysis showed that severity and mortality increase by about 9 and 13%, respectively, for each one-year increase in age (*p* < 0.001). Even after adjustment for age and sex, or age, sex, and comorbidities, age remained a risk factor for severity and mortality due to COVID-19. Alsafar et al. also showed that age was a significant risk factor for severity and mortality after adjustment for sex, age, and smoking, or adjustment for sex, age, smoking, and co-morbidities (26). This could be attributed to the decline of immune system function with increasing age. And unexpectedly, Cereda et al. found a positive correlation between serum 25(OH)D levels and mortality after adjustment for several factors, including age, sex, CRP, ischemic heart disease, and severe pneumonia (28). This inconsistency suggests several confounding factors affect the correlation between vitamin D and the risk of COVID-19, so further well-controlled clinical trials are required to solve these contradictions.

The authors also showed that sex was not a risk factor for disease severity. Also, neither sex nor obesity was risk factors for mortality, which was also observed in our study after adjustment (26). In contrast, a systematic review and meta-analysis by Akbar et al. reported that male gender and diabetes affect the correlation between vitamin D deficiency and mortality (27). Vitamin D may play a role in many mechanisms that are thought to contribute to the male-biased poor outcomes of COVID-19. The impact of gender, and this sex difference in risk may be broadly explained by immune system activity, preexisting cardiovascular diseases, and the impact of various habits that are more commonly practiced by males than females, such as smoking and drinking. The Sex hormone-induced immunological variations and increased expression of angiotensin-converting enzyme-2 (ACE 2; coronavirus receptors) in males may also contribute to this impact (29). Gender differences in coagulation may explain why men have a higher risk of thrombotic/thromboembolic events than women, and the cytokine storm role in inducing vascular inflammation and atherosclerosis-related cardiovascular diseases may also vary between males and females (30).

While SARS-CoV-2 stimulates the release of proinflammatory cytokines, vitamin D inhibits the release of some of these molecules (31). Furthermore, vitamin D is known to regulate different thrombotic pathways, either directly or indirectly. It has been proposed that vitamin D supplementation not only reduces the risk of Acute Respiratory Disease Syndrome (ARDS), but it may also help to reduce coagulation abnormalities in critically ill COVID-19 patients (32).

Previous research also suggests that low Testosterone levels in elderly men may enhance the severity of COVID-19 infection and raise the risk of developing ARDS. The relationship between Testosterone, the immune system, and male aging is well recognized, as is the progressive decline in Testosterone levels with age; this may explain why senior male patients have a greater risk of developing ARDS and die (33). Vitamin D supplementation has been shown to increase testosterone levels in previous research. In comparison to baseline readings, the vitamin D supplemented group demonstrated a considerable rise in total testosterone, bioactive testosterone, and free testosterone levels. In comparison, the placebo group showed no significant change in any testosterone level (34). So, the increased risk of mortality among COVID-19 patients in the presence of vitamin D deficiency could be derived from the above-mentioned difference in vitamin D function and effect between males, and females. Furthermore, Vitamin D is also essential for androgen synthesis inside the testicular cells and endogenous testosterone may explain why low 25-OHD levels are associated with distinct risks in men and women, such as the cardiometabolic risk. This is especially crucial for the elderly. However, the specific mechanism through which gender plays a role is unknown (35, 36).

There was a decline in COVID-19 severity and mortality in our cohort by about 10 and 24%, respectively, for each one-unite increase in lymphocyte count. There was also a statistically significant correlation between declined lymphocyte count and vitamin D deficiency (*p* = 0.003). This observation is consistent with the early report by Tan et al., who suggested that lymphopenia could be considered a reliable indicator for poor COVID-19 outcomes (37). Another cross-sectional study in Iran had reported decreased lymphocyte count among COVID-19 patients with vitamin D deficiency compared to patients with vitamin D sufficiency (24). Karahan and Katkat also observed a strong and negative correlation between lymphocyte count and vitamin D deficiency (*r* = −0.348, *P* < 0.001) (27).

Regarding the immune-inflammatory response markers, we found a significant correlation between vitamin D deficiency, decreased platelets and lymphocyte count, and elevated LDH and Fibrinogen levels. Therefore, vitamin D deficiency has a role in the hyper-inflammation state, oxidative stress, acute and severe lung damage, and increased risk of thromboembolism. Furthermore, SARS-CoV-2 mediated endothelial damage interferes with the binding between vitamin D and its receptor leading to further deterioration of the inflammatory state and increasing the disease severity (38). However, Smolders et al. addressed the reverse causality of the correlation between COVID-19 and the circulating 25(OH)D levels. The authors reported a decrease in the circulating 25(OH) D levels resulting from upregulation of the enzyme 25(OH) D1-alpha-hydroxylase due to COVID-19-associated systemic inflammatory response (39).

The mechanism by which vitamin D could interfere with the outcomes of COVID-19 is still unknown. The protective effect of vitamin D during SARS-CoV-2 viral infection could be mediated via different mechanisms, including stimulation of the production of several antimicrobial peptides, including cathelicidin and defensin in respiratory barriers, decreasing inflammation mediated by its tolerogenic effect and induction of T-reg cell and interleukin-10 (IL-10), while inhibiting IL-12, gamma interferon (IFN-γ), tumor necrosis factor-alpha (TNF-α), IL-2, and IL-17, modulation of the renin-angiotensin pathway, and downregulation of ACE-2 (40–42). A previous meta-analysis conducted by Martineau et al. had shown that the protective effect of supplementary vitamin D during respiratory viral infection is beneficial among those with vitamin D deficiency at the time of infection but not among those taking bolus doses (43).

Our study had some limitations, including the small sample size, the predominance of the male gender, the lack of socioeconomic data in our analysis which could affect the dietary habits, demographic, previous use of vitamins supplementations, or amount of sun exposure living altitudes and sufficient clinical history that could alter vitamin D levels and consequently the risk of COVID-19, and also the difference in the cut-off points between the studies, which could have significant implications on the interpretation of data.

## Conclusion

In summary, our study showed a statistically significant correlation between deficient serum 25(OH)D levels (<12 ng/mL) and poor clinical outcomes among COVID-19 patients. Given our findings and vitamin D's safety as well as its broad therapeutic window, public health policies may recommend vitamin D supplementation to improve COVID-19 patient outcomes, particularly among individuals at high risk of SARS-CoV-2 infection and patients with risk factors for poor COVID-19 outcomes and initial vitamin d deficiency. Larger controlled trials are recommended to control possible confounding factors and determine the optimal cut-off values of serum vitamin D levels.

## Data Availability Statement

The raw data supporting the conclusions of this article will be made available by the authors, without undue reservation.

## Ethics Statement

The studies involving human participants were reviewed and approved by Abu Dhabi Health COVID-19 Research Ethics Committee (DOH/CVDC/2020/231). Written informed consent for participation was not required for this study in accordance with the national legislation and the institutional requirements.

## Author Contributions

WH: literature review, conceptualization, project administration, data analysis and interpretation, revising, writing of original and final draft, and sharing in writing the manuscript. AA, HS, SA, OF, KL, SR, MA, WA, SK, and WE: literature review, analysis, interpretation of data, revising the work, editing, final approval of the version to be published, and agreement to be accountable for all aspects of the work. All authors have contributed to the article, and read and agreed to the published version of the manuscript.

## Conflict of Interest

The authors declare that the research was conducted in the absence of any commercial or financial relationships that could be construed as a potential conflict of interest.

## Publisher's Note

All claims expressed in this article are solely those of the authors and do not necessarily represent those of their affiliated organizations, or those of the publisher, the editors and the reviewers. Any product that may be evaluated in this article, or claim that may be made by its manufacturer, is not guaranteed or endorsed by the publisher.
